# Association of Low Emotional and Tangible Support With Risk of Dementia Among Adults 60 Years and Older in South Korea

**DOI:** 10.1001/jamanetworkopen.2022.26260

**Published:** 2022-08-11

**Authors:** Dae Jong Oh, Hee Won Yang, Tae Hui Kim, Kyung Phil Kwak, Bong Jo Kim, Shin Gyeom Kim, Jeong Lan Kim, Seok Woo Moon, Joon Hyuk Park, Seung-Ho Ryu, Jong Chul Youn, Dong Young Lee, Dong Woo Lee, Seok Bum Lee, Jung Jae Lee, Jin Hyeong Jhoo, Jong Bin Bae, Ji Won Han, Ki Woong Kim

**Affiliations:** 1 Department of Psychiatry, Seoul National University College of Medicine, Seoul, South Korea; 2 Department of Psychiatry, SMG-SNU (Seoul National University) Boramae Medical Center, Seoul, South Korea; 3 Department of Neuropsychiatry, Seoul National University Bundang Hospital, Gyeonggido, South Korea; 4 Department of Psychiatry, Yonsei University Wonju Severance Christian Hospital, Wonju, South Korea; 5 Department of Psychiatry, Dongguk University Gyeongju Hospital, Gyeongju, South Korea; 6 Department of Psychiatry, Gyeongsang National University School of Medicine, Jinju, South Korea; 7 Department of Neuropsychiatry, Soonchunhyang University Bucheon Hospital, Bucheon, South Korea; 8 Department of Psychiatry, School of Medicine, Chungnam National University, Daejeon, South Korea; 9 Department of Psychiatry, School of Medicine, Konkuk University, Konkuk University Chungju Hospital, Chungju, South Korea; 10 Department of Neuropsychiatry, Jeju National University Hospital, Jeju, South Korea; 11 Department of Psychiatry, School of Medicine, Konkuk University, Konkuk University Medical Center, Seoul, Korea; 12 Department of Neuropsychiatry, Kyunggi Provincial Hospital for the Elderly, Yongin, South Korea; 13 Department of Neuropsychiatry, Seoul National University Hospital, Seoul, South Korea; 14 Department of Neuropsychiatry, Inje University Sanggye Paik Hospital, Seoul, South Korea; 15 Department of Psychiatry, Dankook University Hospital, Cheonan, South Korea; 16 Department of Psychiatry, Kangwon National University School of Medicine, Chuncheon, South Korea; 17 Department of Brain and Cognitive Science, Seoul National University College of Natural Sciences, Seoul, South Korea

## Abstract

**Importance:**

The association between social support and dementia risk has been debated. Most previous prospective studies have not differentiated the subtypes of social support.

**Objective:**

To examine whether the association between social support and risk of dementia differs by subtype of social support and by sex.

**Design, Setting, and Participants:**

This nationwide prospective cohort study included randomly sampled South Korean adults 60 years or older. The study was launched November 1, 2010, with follow-up every 2 years until November 30, 2020. The 5852 participants who completed the assessment for social support and were not diagnosed as having dementia, severe psychiatric disorders including major depressive disorder, or major neurological disorders at the baseline assessment were included in the analysis.

**Exposures:**

Geriatric psychiatrists administered the structured diagnostic interviews and physical examinations to every participant based on the Korean version of the Consortium to Establish a Registry for Alzheimer Disease (CERAD-K) Assessment Packet Clinical Assessment Battery.

**Main Outcomes and Measures:**

Baseline levels of emotional and tangible support using the Medical Outcomes Survey Social Support Survey.

**Results:**

Among the 5852 participants (mean [SD] age, 69.8 [6.6] years; 3315 women [56.6%]; mean [SD] follow-up duration, 5.9 [2.4] years), 237 (4.0%) had incident all-cause dementia and 160 (2.7%) had incident Alzheimer disease (AD) subtype of dementia. Compared with women who reported having emotional support, those with low emotional support had almost a 2-fold higher incidence of all-cause dementia (18.4 [95% CI, 13.6-23.2] vs 10.7 [95% CI, 9.0-12.5] per 1000 person-years) and AD (14.4 [95% CI, 10.2-18.6] vs 7.8 [95% CI, 6.3-9.3] per 1000 person-years). Adjusted Cox proportional hazard analysis revealed that low emotional support was associated with increased risk of all-cause dementia (hazard ratio, 1.61 [95% CI, 1.10-2.36]; *P* = .02) and AD (hazard ratio, 1.66 [95% CI, 1.07-2.57]; *P* = .02) only in women. Low tangible support was not associated with a risk of all-cause dementia or AD regardless of sex.

**Conclusions and Relevance:**

The findings of this cohort study suggest that older women with low emotional support constitute a population at risk for dementia. The level of emotional support should be included in risk assessments of dementia.

## Key Points

**Question:**

Are low levels of social support associated with risk of dementia in older adults?

**Findings:**

In this cohort study of 5852 adults 60 years and older, low emotional support was associated with an increased risk of dementia; however, low tangible support was not. Low emotional support was associated with a 61% increased risk of all-cause dementia and a 66% increased risk of Alzheimer disease in women but not in men.

**Meaning:**

These findings suggest that older women with low emotional support should be considered a vulnerable population at risk for dementia and be provided with sufficient empathetic understanding and listening as well as material aid or behavioral assistance.

## Teaser

This cohort study compares the association between low levels of emotional and tangible support and risk of dementia among adults 60 years and older in South Korea.

## Introduction

Social support, a qualitative aspect of social relationships, affects physical and mental health via behavioral, psychological, and physiological pathways; high social support promotes health-promoting behaviors, self-efficacy, adaptive coping styles, and stress-buffering while also modulating hypothalamic-pituitary-adrenal axis, cardiovascular reactivity, and the immune system.^1^ Low social support was associated with various adverse health outcomes such as coronary heart disease,^2^ depression,^3^ and mortality.^4^

Social isolation in late life has been accepted as a potentially modifiable risk factor for dementia.^5^ Social isolation could be determined by the level of social support that reflects the quality and function of social relationships.^1^ However, the association of social support with risk of dementia has been debated. Low social support has been associated with risk of dementia in some prospective studies^6,7,8,9^ but not others.^10,11,12,13^ The conflicting results of these studies might be attributable, at least in part, to a limited evaluation of social support. First, most of the previous prospective studies did not differentiate the subtypes of social support; rather, they evaluated the level of social support using only a single-item questionnaire with categories such as “being satisfied with relationships,” “getting help from others,” or “being understood by others”^6,7,8,10,11,12^ or using multiple-item questionnaires without differentiation of the subtypes.^13^ However, the association of social support with the risk for dementia may be different by its subtypes because neural substrates of social support differed by their subtypes.^14^ The 2 major subtypes of social support include emotional support, which provides empathy, caring, or understanding, and tangible or instrumental support, which provides help, aid, or assistance with tangible needs.^1^ In a prospective study from Japan,^9^ emotional support from friends or neighbors was associated with reduced risk of dementia, but tangible support from friends or neighbors was not associated with the risk of dementia in older adults with disabilities. A replication study is needed to clarify whether association of social support with dementia risk differed by the subtypes of social support in healthy older adults. Furthermore, most previous studies have not considered the effect of sex on the association between social support and the risk of dementia. Because women have been found to be more dependent on emotional support from a widespread social network than men during their lifetimes,^15^ the level of each subtype of social support and its association with the risk for dementia may differ by sex.

In this prospective cohort study of a representative population of older South Korean adults, we compared the associations of emotional and tangible support with risk of dementia. In addition, we examined whether these associations with risk of dementia are dimorphic by sex.

## Methods

### Study Design, Setting, and Participants

The Korean Longitudinal Study on Cognitive Aging and Dementia (KLOSCAD) is a population-based prospective cohort study of older adults.^16^ Based on the 2010 national residential rosters, the KLOSCAD randomly sampled 6818 nationwide representative South Korean adults 60 years or older. Since the baseline assessment period from November 1, 2010, to October 31, 2012, follow-up assessments have been performed at 2-year intervals (first follow-up period from November 1, 2012, to October 31, 2014; second, from November 1, 2014, to October 31, 2016; third, from November 1, 2016, to October 31, 2018; and fourth, from January 1, 2019, to November 30, 2020). For the present analyses, we included 5852 participants from the baseline assessments after excluding those who were diagnosed as having dementia (n = 408), severe psychiatric disorders including major depressive disorder (n = 323), or major neurological disorders (n = 15) or those who did not complete the assessment for social support (n = 220). Among the 5852 participants, 4603 (78.7%) completed the first follow-up assessments; 3783 (64.6%), the second; 3097 (52.9%), the third; and 2306 (39.4%), the fourth.

All the participants were fully informed about the study protocol and provided written informed consent. This study was approved by the institutional review board of the Seoul National University Bundang Hospital. We followed the Strengthening the Reporting of Observational Studies in Epidemiology (STROBE) reporting guideline for cohort studies.

### Assessments of Social Support

We evaluated the level of perceived emotional and tangible social support using the Medical Outcomes Survey social support survey.^17^ The level of each subtype of social support is rated by the sum of the scores of 4 items. For emotional support, we asked how often someone was available “who you can count on to listen to you,” “to confide in,” “to share your worries with,” and “who understands your problems.” For tangible support, we asked how often someone was available “to help you if you confined to bed,” “to take you to the doctor,” “to prepare your meals,” and “to help with daily chores.” Each item was rated on a Likert scale from 1 (none of the time) to 5 (all of the time). The mean (SD) scores of the total 5852 participants were 15.1 (4.3) for emotional support and 16.3 (4.0) for tangible support. The scores of emotional and tangible supports were positively correlated (Pearson *r* = 0.53; *P* < .001). In the present study, we defined *low emotional support* (LES) as an emotional support score below the 25th percentile among the participants at baseline assessment and *low tangible support* (LTS) as a tangible support score below the 25th percentile among participants in the baseline assessment.^18^

### Assessment of Dementia

In the baseline and all follow-up assessments of the KLOSCAD cohort, experienced geriatric psychiatrists (D.J.O., H.W.Y., T.H.K., K.P.K., B.J.K., S.G.K., J.L.K., S.W.M., J.H.P., S.-H.R., J.C.Y., D.Y.L., D.W.L., S.B.L., J.J.L., J.H.J., J.B.B., J.W.H., and K.W.K.) administered the structured diagnostic interviews and physical examinations to every participant based on the Korean version of the Consortium to Establish a Registry for Alzheimer Disease (CERAD-K) Assessment Packet Clinical Assessment Battery.^19^ Trained neuropsychologists performed standardized neuropsychological tests using CERAD-K Neuropsychological Assessment Battery,^20^ Digit Span Forward and Backward tests, and Frontal Assessment Battery.^21^ Research nurses collected blood samples and conducted laboratory tests that consisted of complete blood cell counts, chemistry panels, serologic tests for syphilis, thyroid function tests, serum vitamin B_12_ and folate levels, and apolipoprotein E genotyping. Through regular diagnostic consensus meetings, a panel of geriatric psychiatrists (D.J.O., H.W.Y., J.W.H., and K.W.K.) confirmed the diagnosis of dementia by the *Diagnostic and Statistical Manual of Mental Disorders* (Fourth Edition) criteria^22^ and mild cognitive impairment by the consensus diagnostic criteria of the International Working Group.^23^ The panel diagnosed the Alzheimer disease (AD) subtype of dementia according to the criteria of the National Institute of Neurological and Communicative Disorders and Stroke and the Alzheimer Disease and Related Disorders Association.^24^

### Assessments of Covariates

Trained research nurses assessed sociodemographic factors (age, sex, educational level), health-promoting or -aggravating behaviors (current alcohol consumption, smoking, and the level of physical activities), burden of comorbidities, depressive symptoms, and economic status. We defined the low level of physical activities as less than 600 metabolic equivalent task minutes per week of exercises,^25^ high comorbidities as 5 points or greater on the Cumulative Illness Rating Scale score,^26^ depression as 16 points or greater on the Geriatric Depression Scale score,^27^ and economic disadvantage as being covered by the National Medicaid insurance.

In face-to-face interviews, research nurses evaluated the marital status, presence of cohabitants, and current occupational status as indicators for the social network of each participant. They also evaluated the total amount of social activities (mean hours per month) such as familial gatherings, religious and peer group connections, and volunteer activities during the past year.

### Statistical Analysis

We calculated the age- and sex-adjusted prevalence and incidence of low social support by direct standardization method using the 2010 National Census data. We compared the baseline characteristics of participants according to sex using a χ^2^ test for categorical variables and an unpaired *t* test for continuous variables. We compared the sex-adjusted characteristics by the level of social support using logistic regression and analysis of covariance. We then calculated the age- and sex-adjusted incidence rates of all-cause dementia and AD by sex and the subtype of support. We compared the incidence of all-cause dementia and AD by sex and the subtype of support using the χ^2^ test. To examine whether LES and LTS at baseline assessment are associated with the risks of incident all-cause dementia and AD, we used Cox proportional hazards analyses adjusted for age, sex, educational level, alcohol consumption, smoking, physical activity, comorbidities, depression, economic status, marital status, cohabitants, occupation, and social activities. As sensitivity analyses, we performed the Cox proportional hazards analyses by entering social support of increasing strata (<25th, 25th-75th, and >75th percentiles) and continuous variables. We also used Cox proportional hazards analyses entering the interactions between sex and each type of social support as independent variables. All statistical analyses were performed using SPSS Statistics, version 19.0 (IBM Corporation). Two-sided *P* < .05 indicated statistical significance.

## Results

Among the 5852 participants in the baseline assessment (2537 men [43.3%] and 3315 women [56.6%]; mean [SD] age, 69.8 [6.6] years), 1167 (508 men and 659 women) had LES and 1325 (361 men and 964 women) had LTS. Five hundred fifty participants (174 men and 376 women) had both LES and LTS. Among the 4685 participants without LES at baseline assessment (mean [SD] follow-up duration, 5.3 [2.5] years), 829 (329 men and 500 women) had incident LES at 1 or more follow-up assessments. Among the 4527 participants without LTS at baseline assessment (mean [SD] follow-up duration, 5.1 [2.5] years), 947 (289 men and 658 women) had incident LTS at 1 or more follow-up assessments (Table 1). When we analyzed men and women separately, the prevalence of LES was comparable between men and women, whereas the incidence of LES was higher in women than in men (44.9 [95% CI, 41.1-48.6] vs 38.8 [95% CI, 34.7-42.9] per 1000 person-years; *P* = .04). Both the prevalence (29.1 [95% CI, 27.6-30.7] vs 14.2 [95% CI, 12.8-15.6] per 1000 person-years) and incidence (73.1 [95% CI, 67.8-78.4] vs 30.3 [95% CI, 26.9-33.8] per 1000 person-years) of LTS were more than 2-fold higher in women than in men (*P* < .001) (Table 1).

**Table 1.** Prevalence and Incidence Estimates of Low Social Support by Sex and Subtype of Support^a^

Participants	Low emotional support		Low tangible support	
	Prevalence (95% CI) per 1000 person-years	Incidence (95% CI) per 1000 person-years	Prevalence (95% CI) per 1000 person-years	Incidence (95% CI) per 1000 person-years
All	20.4 (19.4-21.4)	42.3 (39.5-45.0)	22.7 (21.7-23.8)	54.8 (51.5-58.0)
Men	20.0 (18.4-21.5)	38.8 (34.7-42.9)	14.2 (12.8-15.6)	30.3 (26.9-33.8)
Women	20.7 (19.3-22.1)	44.9 (41.1-48.6)	29.1 (27.6-30.7)	73.1 (67.8-78.4)

^a^ Adjusted by the direct standardization method based on 2010 National Census data.

As summarized in Table 2, participants with LES and those with LTS were more economically disadvantaged; had less physical activity; were more likely to be widowed, divorced, or single and living alone; and were more likely to have comorbidities and depression than those without LES and LTS. Participants with LES were older (mean [SD], 70.9 [6.9] vs 69.5 [6.4] years) and less educated (mean [SD], 7.0 [5.3] vs 8.6 [5.2] years) and had more comorbidities (561 of 1167 [48.1%] vs 1948 of 4685 [41.6%]) and fewer social activities (mean [SD], 27.6 [47.0] vs 37.7 [78.8] h/mo) than those without LES. Participants with LTS were more likely to be women (964 of 1325 [72.7%] vs 2351 of 4527 [51.9%]), less likely to smoke (133 of 1325 [10.0%] vs 537 of 4527 [11.9%]), and more likely to have no current occupation (1005 of 1325 [75.8%] vs 3001 of 4527 [66.3%]) compared with those without LTS (Table 2).

**Table 2.** Baseline Characteristics of Study Participants

Characteristic	Stratified by sex			Stratified by level of support					
				Emotional			Tangible		
	Men (n = 2537)	Women (n = 3315)	*P* value^a^	Not low (n = 4685)	Low (n = 1167)^b^	*P* value^c^	Not low (n = 4527)	Low (n = 1325)^b^	*P* value^c^
Women	NA	NA	NA	2656 (56.7)	659 (56.5)	.89	2351 (51.9)	964 (72.7)	<.001
Age, mean (SD), y	69.4 (6.3)	70.1 (6.7)	<.001	69.5 (6.4)	70.9 (6.9)	<.001	69.7 (6.5)	70.1 (6.7)	.15
Educational level, mean (SD), y	10.6 (4.9)	6.5 (4.9)	<.001	8.6 (5.2)	7.0 (5.3)	<.001	8.4 (5.3)	7.6 (5.2)	.78
Current alcohol consumption^d^	703 (27.7)	63 (1.9)	<.001	608 (13.0)	158 (13.5)	.63	645 (14.2)	121 (9.1)	.79
Current smoking	585 (23.1)	85 (2.6)	<.001	518 (11.1)	152 (13.0)	.05	537 (11.9)	133 (10.0)	.003
Low level of physical activities^e^	959 (37.8)	1855 (55.9)	<.001	2177 (46.5)	637 (54.6)	<.001	2100 (46.4)	714 (53.9)	.01
High level of comorbidities^f^	1163 (45.8)	1346 (40.6)	<.001	1948 (41.6)	561 (48.1)	<.001	1917 (42.3)	592 (44.7)	.02
Depression^g^	347 (13.7)	719 (21.7)	<.001	612 (13.1)	454 (38.9)	<.001	643 (14.2)	423 (31.9)	<.001
Economic disadvantage^h^	73 (2.9)	175 (5.3)	<.001	135 (2.9)	113 (9.7)	<.001	113 (2.5)	135 (10.2)	<.001
Widowed, divorced, or single	232 (9.1)	1404 (42.3)	<.001	1205 (25.7)	431 (36.9)	<.001	1057 (23.3)	579 (43.7)	<.001
Living alone	145 (5.7)	669 (20.2)	<.001	581 (12.4)	233 (20.0)	<.001	467 (10.3)	347 (26.2)	<.001
No current occupation	1387 (54.7)	2619 (79.0)	<.001	3211 (68.5)	795 (68.1)	.80	3001 (66.3)	1005 (75.8)	.001
Social activities, mean (SD), h/mo^i^	32.0 (60.0)	38.5 (82.5)	<.001	37.7 (78.8)	27.6 (47.0)	<.001	36.5 (72.2)	33.0 (78.4)	.03

Abbreviation: NA, not applicable.

^a^ Calculated using a χ^2^ test for categorical variables and an unpaired *t* test for continuous variables.

^b^ Each score of the Medical Outcomes Study social support survey lower than 25th percentile of participants was categorized as a low level of social support.

^c^ Sex-adjusted *P* values calculated by logistic regression for categorical variables and analysis of covariance for continuous variables.

^d^ Indicates greater than 7 standard U/wk within 1 year.

^e^ Indicates less than 600 metabolic equivalent task minutes per week.

^f^ Indicates Cumulative Illness Rating Scale scores of 5 points or greater.

^g^ Indicates Geriatric Depression Scale scores of 16 points or greater.

^h^ Indicates covered by National Medicaid.

^i^ Includes familial gatherings, religious, peer group, or volunteer activities.

Among the 5852 participants who did not have dementia at baseline assessment, 237 (4.0%) were diagnosed as having incident dementia during the follow-up period (mean [SD] follow-up duration, 5.9 [2.4] years). Among the 237 participants with incident dementia, 160 (48.9%; 2.7% of all participants) were diagnosed as having AD. The incidence of all-cause dementia was higher in participants with vs without LES among both men (10.7 [95% CI, 6.6-14.9] vs 6.8 [95% CI, 5.1-8.4] per 1000 person-years) and women (18.4 [95% CI, 13.6-23.2] vs 10.7 [95% CI, 9.0-12.5] per 1000 person-years). The incidence of AD, however, was higher in participants with than without LES in women only (14.4 [95% CI, 10.2-18.6] vs 7.8 [95% CI, 6.3-9.3] per 1000 person-years). The incidence of all-cause dementia was comparable between participants with and without LTS among both men and women. The incidence of AD, however, was higher in participants with than without LTS in women only (11.1 [95% CI, 8.2-14.0] vs 8.4 [95% CI, 6.7-10.0] per 1000 person-years) (Table 3).

**Table 3.** Adjusted Incidence Estimates of All-Cause Dementia and Alzheimer Disease by Sex and Subtype of Social Support

Stratification	All-cause dementia		Alzheimer disease	
	Incidence (95% CI)^a^	*P* value^b^	Incidence (95% CI)^a^	*P* value^b^
**All participants**				
By emotional support				
Not low	9.0 (7.8-10.3)	<.001	6.1 (5.1-7.1)	<.001
Low	15.1 (11.9-18.3)		10.8 (8.0-13.5)	
By tangible support				
Not low	10.2 (8.8-11.5)	.08	6.6 (5.5-7.6)	.002
Low	7.8 (5.7-9.9)		8.5 (6.4-10.8)	
**Men**				
By emotional support				
Not low	6.8 (5.1-8.4)	.04	3.9 (2.6-5.1)	.13
Low	10.7 (6.6-14.9)		6.0 (2.9-9.1)	
By tangible support				
Not low	7.4 (5.7-9.0)	.50	4.1 (2.9-5.4)	.59
Low	8.9 (4.3-13.4)		5.1 (1.6-8.6)	
**Women**				
By emotional support				
Not low	10.7 (9.0-12.5)	<.001	7.8 (6.3-9.3)	<.001
Low	18.4 (13.6-23.2)		14.4 (10.2-18.6)	
By tangible support				
Not low	12.3 (10.2-14.3)	.26	8.4 (6.7-10.0)	.01
Low	7.0 (4.7-9.3)		11.1 (8.2-14.0)	

^a^ Adjusted by the direct standardization method based on the 2010 National Census data; presented as the number of incidence cases per 1000 person-years.

^b^ Calculated by χ^2^ test.

Cox proportional hazards models that adjusted for age, sex, educational attainment, alcohol consumption, smoking, physical activity, comorbidities, depression, economic status, marital status, cohabitants, occupation, and social activities showed that LES was associated with a 42% increased risk of all-cause dementia (hazard ratio [HR], 1.42 [95% CI, 1.04-1.93]) and a 45% increased risk of AD (HR, 1.45 [95% CI, 1.00-2.11]). When we analyzed men and women independently, LES was associated with a 61% increased risk of all-cause dementia (HR, 1.61 [95% CI, 1.10-2.36]) and a 66% increased risk of AD (HR, 1.66 [95% CI, 1.07-2.57]) in women only. In both sexes, LTS was not associated with the risks of all-cause dementia and AD (Table 4). We also found a significant association LES with risk of all-cause dementia and AD in women when we entered social support of increasing strata (HR, 2.47 [95% CI, 1.28-4.76] in all-cause dementia; HR, 2.26 [95% CI, 1.06-4.81] in AD) and continuous variables (HR, 0.93 [95% CI, 0.89-0.97] in all-cause dementia; HR, 0.92 [95% CI, 0.88-0.97] in AD) (eTable 1 in the Supplement). The interaction between LES and sex was significant only when the level of social support was stratified into low, middle, and high levels of support (HR, 3.10 [95% CI, 1.37-7.04] in all-cause dementia; HR, 3.12 [95% CI, 1.11-8.79] in AD) (eTable 2 in the Supplement).

**Table 4.** Low Level of Social Support and Risk of Incident All-Cause Dementia and Alzheimer Disease by Subtype of Social Support^a^

Participants	All-cause dementia		Alzheimer disease	
	HR (95% CI)	*P* value^b^	HR (95% CI)	*P* value^b^
**All (n = 5852)**				
Low emotional support	1.42 (1.04-1.93)	.03	1.45 (1.00-2.11)	.05
Low tangible support	0.79 (0.57-1.09)	.16	0.99 (0.69-1.44)	.97
**Men (n = 2537)**				
Low emotional support	1.25 (0.74-2.09)	.40	1.28 (0.64-2.53)	.49
Low tangible support	1.00 (0.52-1.93)	>.99	1.14 (0.49-2.65)	.77
**Women (n = 3315)**				
Low emotional support	1.61 (1.10-2.36)	.02	1.66 (1.07-2.57)	.02
Low tangible support	0.82 (0.57-1.19)	.29	1.08 (0.72-1.64)	.71

Abbreviation: HR, hazard ratio.

^a^ Cox proportional hazards models were adjusted for age, sex, educational level, alcohol consumption, smoking, physical activity, comorbidities, depressive symptoms, economic status, marital status, cohabitants, occupation, and social activities. Each score of the Medical Outcomes Study social support survey lower than 25th percentile of participants was defined as the low level of social support.

^b^ *P* < .05 in total population and *P* < .025 with Bonferroni correction in sex-stratified analysis indicated significance.

## Discussion

In a previous study using claims data for older adults with disabilities from a long-term care insurance system in Japan,^9^ emotional support was associated with a 15% lower risk of incident dementia by 15% in women and an 18% lower risk of incident dementia in men, whereas tangible support did not change the risk of incident dementia. However, to our knowledge, the differential association between emotional and tangible support with the risk of dementia has not been investigated in older adults. This study demonstrated that LES was associated with increased risks of all-cause dementia and AD by approximately 40% among older women but not men. However, LTS was not associated with the risks of all-cause dementia and AD in both sexes. Emotional support—that is, the feeling of being understood, cared for, reassured, and provided with chances for emotional expression—may protect against the development of dementia by buffering the effect of stressful events.^28^ Chronic repeated stress may lead to synaptic suppression and dendritic remodeling in AD-related brain regions such as the hippocampus, amygdala, and prefrontal cortex by inducing glucocorticoid toxicity through hypothalamic-pituitary-adrenal axis hyperactivation.^29^ In a previous study,^30^ the association between LES and cognitive decline was mediated by hippocampal atrophy. In contrast to the emotional support that may cover a wide range of stressful events, tangible support may buffer stress from a specific need associated with a certain event.^28^

This study also found that the association of LES with the risk of dementia differed by sex. Because women have been shown to be more dependent on a wide range of emotional support resources under stressful situations compared with men,^15^ the shrinkage of a supportive social network may put women at greater risk for dementia than men, which may contribute at least in part to the sex-dimorphic association of LES with risk of dementia. Compared with men who depend on close relationships such as a few family members, women depend on larger and more variable social networks.^31,32^ With advancing age, the size of social networks other than family decreases faster than that of a family network.^33^ Therefore, women may become more vulnerable to LES and the subsequent risk of AD as they grow older. However, the role of sex in the association between LES and dementia risk should be interpreted cautiously, because some of our interaction analyses were not statistically significant.

Previous epidemiological studies^34,35^ have found a higher incidence of AD in women than in men, which may be the result of sex differences in risk factors.^36^ Furthermore, women are known to be more vulnerable to AD-related pathology (eg, β-amyloid and tau deposition) than men.^37^ The present study found that the sex-specific association was independent of covariates as well as the higher incidence of LES in women than in men. Therefore, along with the other sex-specific risk factors, the sex-dimorphic association of LES with risk of AD may contribute to the high incidence of AD in women. Further investigation is needed to clarify the role of LES in the association between sex and risk of dementia.

The findings of the present study suggest that previous conflictive findings regarding the association of social support with dementia risk may be attributable to the partial or ambiguous assessment of social support. These conflicts become more distinct when these previous studies are classified by subtype of social support. In line with the results of the present study, studies that only evaluated the emotional aspect of social support (eg, being understood or listened to by others)^6,8,9^ reported a significant protective association of social support with risk of dementia, whereas studies that only examined the tangible aspect of social support (eg, getting help or being cared for by others when sick)^9,12^ reported no such association. Meanwhile, studies using single- or multiple-item questionnaires without clear distinctions between emotional and tangible subtypes (eg, being satisfied with relationships or having good relationships)^7,10,11,13^ inconsistently reported associations between social support and dementia risk. By evaluating and differentiating multiple subtypes of support using comprehensive and structured assessment tools, the findings of the present study suggest that the association between social support and risk of dementia largely depends on the subtype of social support.

Our findings further suggest that the cognitive function of women with LES should be tracked carefully in primary care and outpatient clinics. In terms of public health, we also suggest that older women with LES should be considered a population at risk for dementia and should therefore be provided with emotional support from their community. Few studies have examined the effectiveness of enhancing social activities against cognitive decline in cognitively normal older adults. Two studies that used activities focusing on cognitive and physical functions but not emotional support found no significant improvement in cognition.^38,39^ Another study that used activities strengthening emotional support found a limited but significant improvement in cognition.^40^ Future investigations are needed to develop intervention strategies focusing on emotional support and to identify the effectiveness of those strategies to prevent dementia in women.

### Limitations

Several limitations should be acknowledged in this study. First, we evaluated the level of perceived social support but not the level of actual social support received. Although a previous study^17^ reported that perceived social support could reflect the degree of availability of support more accurately than the support received, the possible discrepancies between perceived and received social support should be noted. Second, although we adjusted covariates such as marital status, cohabitants, occupation, and frequency of participation in social activities that may be related to social relationships, we did not directly evaluate the sources of social support and the size of social networks. Third, our findings should be generalized with caution because social relationships and their association with the risk for dementia may differ by race and ethnicity, culture, and social structures. Our findings should be replicated in different settings or countries with various social environments. Fourth, this study is also subject to an attrition bias. Additionally, whether LES was the prodromal condition or a risk factor for dementia was unclear because the follow-up duration by the onset of dementia was relatively short.

## Conclusions

To our knowledge, this cohort study is the first to demonstrate that the association between social support and risk of dementia is differentiated by the subtypes of social support and sex. The level of perceived emotional support is worthy of being included in the risk assessment of dementia in older adults, particularly in women.

## Notes

Supplement. **eTable 1.** Level of Each Type of Social Support and the Risks of Incident All-Cause Dementia and Alzheimer Disease **eTable 2.** Level of Each Type of Social Support and Their Interactions With Sex and the Risks of Incident All-Cause Dementia and Alzheimer Disease
