# Supplementary material for: Association of Low Emotional and Tangible Support With Risk of Dementia Among Adults 60 Years and Older in South Korea
Source: JAMA Netw Open. 2022 Aug 11;5(8):e2226260. doi: 10.1001/jamanetworkopen.2022.26260 (PMC9372780; doi:10.1001/jamanetworkopen.2022.26260)
Supplement: Supplement. — eTable 1. Level of Each Type of Social Support and the Risks of Incident All-Cause Dementia and Alzheimer Disease eTable 2. Level of Each Type of Social Support and Their Interactions With Sex and the Risks of Incident All-Cause Dementia and Alzheimer Disease [file jamanetwopen-e2226260-s001.pdf]

Supplementary Online Content

Oh DJ, Yang HW, Kim TH, et al. Association of low emotional and tangible support with risk of dementia among adults 60 years and older in South Korea. *JAMA Netw Open*. 2022;5(8):e2226260. doi:10.1001/jamanetworkopen.2022.26260

eTable 1. Level of Each Type of Social Support and the Risks of Incident All-Cause Dementia and Alzheimer Disease

eTable 2. Level of Each Type of Social Support and Their Interactions With Sex and the Risks of Incident All-Cause Dementia and Alzheimer Disease

This supplementary material has been provided by the authors to give readers additional information about their work.

© 2022 Oh DJ et al. *JAMA Network Open*.

eTable 1. Level of Each Type of Social Support and the Risks of Incident All-Cause Dementia and Alzheimer Disease

	All-cause dementia			Alzheimer's disease	
	HR (95% CI)	*p*		HR (95% CI)	*p*
Low or middle vs. high (reference)					
Total (n = 5,852)					
Low emotional support	1.72 (1.07 – 2.77)	0.03		1.70 (0.94 – 3.08)	0.08
Middle emotional support	1.30 (0.85 – 1.99)	0.23		1.24 (0.73 – 2.13)	0.43
Low tangible support	0.79 (0.49 – 1.28)	0.34		1.11 (0.62 – 1.99)	0.72
Middle tangible support	1.03 (0.73 – 1.46)	0.85		1.30 (0.83 – 2.03)	0.25
Men (n = 2,537)					
Low emotional support	1.00 (0.48 – 2.07)	1.00		0.93 (0.34 – 2.54)	0.89
Middle emotional support	0.85 (0.46 – 1.60)	0.62		0.84 (0.35 – 1.99)	0.69
Low tangible support	1.03 (0.40 – 2.64)	0.95		1.76 (0.55 – 5.68)	0.34
Middle tangible support	0.75 (0.44 – 1.28)	0.29		0.94 (0.45 – 1.96)	0.87
Women (n = 3,315)					
Low emotional support	2.47 (1.28 – 4.76)	0.01		2.26 (1.06 – 4.81)	0.04
Middle emotional support	1.71 (0.93 – 3.14)	0.08		1.43 (0.70 – 2.92)	0.32
Low tangible support	0.97 (0.53 – 1.81)	0.93		1.36 (0.65 – 2.82)	0.42
Middle tangible support	1.45 (0.89 – 2.36)	0.14		1.79 (0.97 – 3.30)	0.06
Continuous variable					
Total (n = 5,852)					
Emotional support	0.94 (0.91 – 0.97)	<.001		0.93 (0.89 – 0.97)	<.001
Tangible support	1.03 (0.99 – 1.07)	0.15		1.01 (0.97 – 1.05)	0.72
Men (n = 2,537)					
Emotional support	0.98 (0.92 – 1.04)	0.50		0.96 (0.89 – 1.04)	0.30
Tangible support	1.03 (0.96 – 1.11)	0.45		1.01 (0.92 – 1.11)	0.83
Women (n = 3,315)					
Emotional support	0.93 (0.89 – 0.97)	<.001		0.92 (0.88 – 0.97)	0.001
Tangible support	1.02 (0.97 – 1.06)	0.46		1.00 (0.95 – 1.05)	0.85

HR, hazard ratio; CI, confidence interval

Cox proportional hazard models were adjusted for age, gender, education, drinking, smoking, physical activity, comorbidities, depressive symptoms, economic status, marital status, cohabitants, occupation, and social activities; The score of the emotion-related support is the summation of the scores of the emotional support, affectionate support, and positive social interaction in the Medical Outcomes Study Social Support Survey; Each score of the Medical Outcomes Study Social Support Survey lower than 25th percentile of participants was defined as the low level of social support

© 2022 Oh DJ et al. *JAMA Network Open*.

eTable 2. Level of Each Type of Social Support and Their Interactions With Sex and the Risks of Incident All-Cause Dementia and Alzheimer Disease

	All-cause dementia			Alzheimer's disease	
	HR (95% CI)	*p*		HR (95% CI)	*p*
Low vs. not low (reference)					
Low emotional support*women	1.39 (0.77 – 2.50)	0.16		1.55 (0.73 – 3.27)	0.26
Low tangible support*women	1.22 (0.62 – 2.43)	0.57		1.67 (0.69 – 4.02)	0.25
Low or middle vs. high (reference)					
Low emotional support*women	3.10 (1.37 – 7.04)	0.007		3.12 (1.11 – 8.79)	0.03
Low tangible support*women	1.73 (0.65 – 4.63)	0.27		1.73 (0.53 – 5.67)	0.36
Middle emotional support*women	2.84 (1.33 – 6.06)	0.007		2.41 (0.93 – 6.26)	0.07
Middle emotional support*women	2.67 (1.43 – 4.96)	0.002		2.67 (1.20 – 5.98)	0.02
Continuous variable					
Emotional support*women	0.95 (0.89 – 1.01)	0.08		0.95 (0.88 – 1.02)	0.16
Tangible support*women	0.95 (0.88 – 1.02)	0.15		0.94 (0.85 – 1.02)	0.15

HR, hazard ratio; CI, confidence interval

Cox proportional hazard models were adjusted for emotional and tangible support, gender, age, education, drinking, smoking, physical activity, comorbidities, depressive symptoms, economic status, marital status, cohabitants, occupation, and social activities; Each score of the Medical Outcomes Study Social Support Survey < 25th percentile, 25th - 75th percentile, and > 75th percentile of participants were defined as the low, middle, and high level of social support

© 2022 Oh DJ et al. *JAMA Network Open*.
